# Longitudinal changes in physical activity during and after the first national lockdown due to the COVID-19 pandemic in England

**DOI:** 10.1038/s41598-021-97065-1

**Published:** 2021-09-02

**Authors:** Feifei Bu, Jessica K. Bone, John J. Mitchell, Andrew Steptoe, Daisy Fancourt

**Affiliations:** 1 Department of Behavioral Science and Health, University College London, 1-19 Torrington Place, London, WC1E 7HB UK; 2 Institute of Epidemiology and Public Health, University College London, London, UK

**Keywords:** Risk factors, Public health

## Abstract

Recent studies have shown reduced physical activity at early stages of the COVID-19 pandemic. However, there is a lack of investigation on longitudinal changes in physical activity beyond lockdowns and stay-at-home orders. Moreover, it is unclear if there is heterogeneity in physical activity growth trajectories. This study aimed to explore longitudinal patterns of physical activity and factors associated with them. Data were from the UCL COVID-19 Social Study. The analytical sample consisted of 35,915 adults in England who were followed up for 22 weeks from 24th March to 23rd August 2020. Data were analysed using growth mixture models. Our analyses identified six classes of growth trajectories, including three stable classes showing little change over time (62.4% in total), two classes showing decreasing physical activity (28.6%), and one class showing increasing physical activity over time (9%). A range of factors were found to be associated the class membership of physical activity trajectories, such as age, gender, education, income, employment status, and health. There is substantial heterogeneity in longitudinal changes in physical activity during the COVID-19 pandemic. However, a substantial proportion of our sample showed persistent physical inactivity or decreasing physical activity. Given the well-established link between physical activity and health, persistent or increased physical inactivity is likely to have both immediate and long-term implications for people’s physical and mental health, as well as general wellbeing. More efforts are needed to promote physical activity during the pandemic and beyond.

## Introduction

There is extensive evidence on the positive impacts of physical activity for both physical and mental health. For example, regular physical activity is associated with reduced rates of all-cause mortality, obesity, cancer, cardiovascular disease, stroke, and diabetes, among other physical conditions, as well as depression, anxiety, and other mental health problems^1–4^. Regular physical activity also leads to improvements in cardiorespiratory and muscular fitness, functional health, cognitive function, wellbeing, and quality of life^1,3,5^. However, levels of physical inactivity are rising in many countries^3^. Physical inactivity is the fourth leading risk factor for global mortality^6^, causing 9% of premature mortality worldwide^7^. Physical inactivity is also one of the most important risk factors for chronic diseases globally^8–11^. Even short-term physical inactivity, over a period of 1–4 weeks, has been associated with negative changes in cardiovascular function and increased cardiovascular risk factors^12,13^.

Since December 2019, there has been an outbreak of coronavirus disease (COVID-19). Lockdowns and stay-at-home orders have been announced globally to control the spread of the disease, disrupting people’s usual behaviours. In many countries, this has involved the closure of non-essential businesses, including gyms, outdoor sports amenities, and playgrounds, as well as limits placed on how often people could leave their homes each day. However, how this has affected physical activity levels has been complex. Large cross-sectional surveys in many western countries have suggested decreases in overall physical activity when comparing self-reports of activity levels before to during lockdowns^14–25^. The majority of longitudinal studies to date also show an initial drop in physical activity levels at the start of lockdown restrictions^14,26–31^, as do data from wearable fitness trackers in a number of countries^32–35^. However, some wearable fitness tracker studies suggest that activity levels may not have uniformly decreased across populations^32–35^. Further, following an initial decrease in activity levels as lockdowns first came in, population-level data on the frequency of google searches for terms related to ‘exercise’ compared to ‘television show’ significantly increased in some countries such as the UK even after adjusting for increased interest in television shows^36^. This suggests that the effects of lockdowns and broader social restrictions on physical activity may be more nuanced.

Additionally, studies examining longer-term impacts on physical activity have provided mixed findings on whether physical activity returned to pre-lockdown levels with the initial easing of restrictions in the UK^26,27,30,31^. To our knowledge, no studies have investigated changes in physical activity after further easing of restrictions in the UK in July 2020. The longer term impacts of lockdown measures on physical activity therefore remains unclear, yet it is a crucial question to identify whether short-term disruptions to physical activity behaviours lead to long-term changes that could have adverse effects on health. Moreover, studies to date have assumed one homogeneous trajectory of physical activity, without exploring the heterogeneous patterns of longitudinal changes. This is also vital to understand, as different people may have reacted differently to the pandemic and lockdown measures and therefore be at a higher or lower risk of sustained changes to their physical activity levels. Some studies have been exploring factors likely to influence physical activity levels. Being women, younger, single, a parent, and from an ethnic minority group as well as having poor health, lower education and income, and no access to outside space have been associated with lower physical activity during lockdowns^27,28,31,32,37–43^. There is also some evidence that physical activity habits prior to the onset of the COVID-19 pandemic are associated with changes in physical activity at the start of lockdown^14,26,29,38,44–48^. However, some findings have been inconsistent, and many studies have only examined physical activity at one point early in the pandemic. It therefore remains unclear whether similar factors are associated with the subsequent trajectories of physical activity throughout lockdown and the easing of restrictions.

Consequently, this study set out to examine the heterogeneity in the longitudinal changes of physical activity in England, using a large sample of 35,915 adults tracked across 22 weeks from the 24th March to 23rd August 2020 encompassing a strict lockdown followed by the easing of restrictions. Further, it sought to explore sociodemographic and health-related factors that might be associated with different patterns of longitudinal changes in physical activity. Given the well-established health benefits of physical activity, understanding changes in physical activity habits, and predictors of these changes, is essential for informing healthcare policy in the aftermath of COVID-19.

## Method

### Study design and participants

This study analysed data from the COVID-19 Social Study, a longitudinal study run by University College London (UCL) that focuses on the psychological and social experiences of adults living in the UK during the COVID-19 pandemic. The study commenced on 21st March 2020 and involved weekly online data collection from participants until 23rd August 2020, followed by monthly data collection for the duration of the pandemic. The study did not use a random sample design, but it does contain a heterogeneous sample that was recruited using three primary approaches. First, convenience sampling was used, including promoting the study through existing networks and mailing lists (including large databases of adults who had previously consented to be involved in health research across the UK), print and digital media coverage, and social media. Second, more targeted recruitment was undertaken focusing on (1) individuals from a low-income background, (2) individuals with no or few educational qualifications, and (3) individuals who were unemployed. Third, the study was promoted via partnerships with third sector organisations to vulnerable groups, including adults with pre-existing mental health conditions, older adults, carers, and people experiencing domestic violence or abuse. A full protocol for the study is available online at www.COVIDSocialStudy.org.

To examine trajectories of physical activity in relation to specific lockdown measures, we focused solely on participants who lived in England (N = 56,428). We included participants who had at least three repeated measures between 24th March 2020, the day after the first lockdown started in the UK, and August 23rd 2020, when the survey switched to monthly follow-up and the relevant measure was discontinued. This period encompasses the first national lockdown followed by the easing of restrictions allowing unlimited outdoor exercises (13th May 2020), reopening outdoor gyms and playgrounds (4th July 2020) and indoor gyms and swimming pools (25th July 2020). These criteria provided us with data from 38,917 participants who were followed up for a maximum of 22 weeks. After excluding participants with missing values (8%), our final analytic sample size was 35,915.

### Measures

In the UCL COVID-19 Social Study, participants were asked weekly how long they had spent on the last working day (1) going out for a walk or other gentle physical activity, (2) going out for moderate or high intensity activity (e.g. running, cycling or swimming), (3) exercising inside their home or garden (e.g. doing yoga, weights or other indoor exercise). Responses were recorded on a five-point frequency scale: did not do, < 30 min, 30 min–2 h, 3–5 h and ≥ 6 h. We generated a composite physical activity measure by using the highest response across these three questions, which was recoded into four categories: (1) did not do, (2) < 30 min, (3) 30 min–2 h and (4) ≥ 3 h. For instance, if a participant walked for ≥ 3 h, did high intensity activity for 30 min–2 h, and did not exercise at home, they would be coded as ≥ 3 h. As the physical activity questions referred to the last working day, and the first national lockdown started on 23rd March 2020, we included responses from 24th March 2020 onwards.

A range of socio-demographic and health-related factors were considered as potential predictors of physical activity trajectories. These included gender (women, men), ethnicity (white, ethnic minorities), age groups (18–29, 30–45, 46–59, 60+ years), education (General Certificate of Secondary Education (GCSE) or below, Advanced Level qualifications (A-levels) or equivalent, and university degree or above), household income (< £30,000, ≥ £30,000 per annum), employment status (employed throughout, employed at baseline but lost job during the follow-up, unemployed or economically inactive), living arrangement (living alone, living with others but no children, living with others including children), and area of living (city, large town, small town, rural). Health-related factors were self-reported diagnosis of any long-term physical health condition, including disability (yes, no), and self-reported diagnosis of any long-term mental health condition (yes, no).

### Statistical analysis

Data were analysed using the growth mixture modelling (GMM) approach. The conventional growth modelling approach assumes one homogeneous growth trajectory, allowing individual growth factors to vary randomly around the overall mean. GMM relaxes this assumption and enables researchers to explore different patterns of change (latent trajectory classes)^49^.

We included a polynomial time function to allow for nonlinear growth trajectories informed by the data. Starting with the unconditional GMM, we compared models with different number of classes on the basis of the Bayesian information criterion (BIC) and sample-size adjusted Bayesian information criterion (ABIC), along with the Vuong-Lo-Mendell-Rubin likelihood ratio (LMR-LR) test and Adjusted Lo-Mendell-Rubin likelihood ratio (ALMR-LR) test. After identifying the optimal number of classes, we introduced predictors to explain the observed heterogeneity between classes.

Weights were applied throughout the analyses. The final analytical sample was weighted to the proportions of gender, age, ethnicity and education in the English population obtained from the Office for National Statistics^17^. The main analyses were implemented in Mplus Version 8.

### Ethics approval and consent to participate

The COVID-19 Social Study was approved by the UCL Research Ethics Committee [12467/005].  All methods were performed in accordance with the relevant guidelines and regulations, and all participants gave informed consent.

## Results

### Descriptive

The analytical sample comprised 35,915 participants of whom 75.6% were women, and there was an over-representation of people with a degree or above (70.3%) and an underrepresentation of people from ethinic minority backgrounds (5%; Table 1). Younger adults under 30 years of age were also underrepresented in the sample. After weighting, the sample reflected population proportions, with 51% women, 34.7% participants with a degree or above, 14.5% participants being ethnic minortiy and 19.5% aged under 30.

**Table 1:** Characteristics of the sample (N = 35,915).

	Raw data		Weighted data	
	Percent (%)	N	Percent (%)	N
**Gender**				
Women	75.9	27,245	51.0	18,330
Men	24.1	8670	49.0	17,585
**Ethnicity**				
Minority	5.0	1796	14.5	5223
White	95.0	34,119	85.5	30,692
**Age**				
18–29	7.4	2656	19.5	7012
30–45	29.0	10,431	26.4	9483
46–59	33.0	11,858	24.1	8657
60 +	30.5	10,970	30.0	10,763
**Education**				
GCSE or below	12.9	4636	32.5	11,656
A-levels or equivalent	16.8	6031	32.9	11,807
Degree or above	70.3	25,248	34.7	12,452
**Household income**				
< 30 k	36.7	13,193	46.0	16,519
≥ 30 k	63.3	22,722	54.0	19,396
**Employment status**				
Employed	55.7	19,988	49.0	17,587
Employed to unemployed	10.4	3724	10.1	3636
Unemployed/economically inactive	34.0	12,203	40.9	14,692
**Living status**				
Alone	19.8	7100	18.3	6577
With others (not children)	53.2	19,106	56.2	20,168
With others (including children)	27.0	9709	25.5	9169
**Area of living**				
City	35.4	12,717	34.8	12,503
Large town	19.1	6859	21.8	7817
Small town	24.8	8916	24.4	8770
Rural area	20.7	7423	19.0	6825
**Long term physical illness**				
Yes	39.2	14,086	40.8	14,651
No	60.8	21,829	59.2	21,264
**Long term mental illness**				
Yes	18.3	6578	20.0	7186
No	81.7	29,337	80.0	28,729

**Figure 1:**
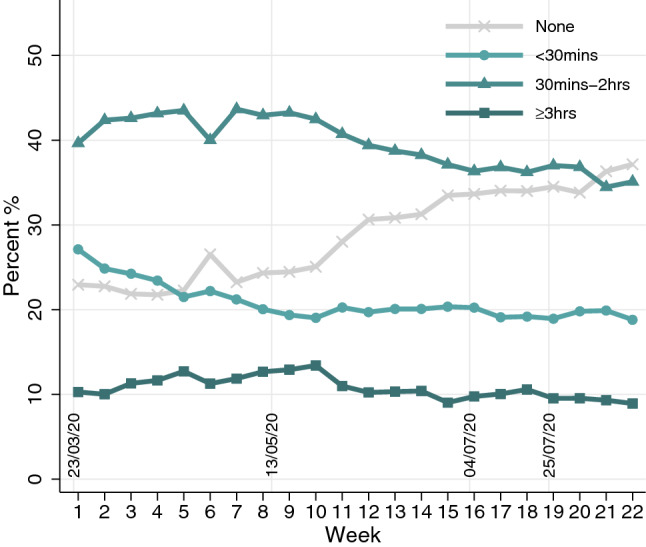
Descriptive changes of physical activity over 22 weeks. On March 23, 2020, the first lockdown commenced in England. All non-essential businesses, including gyms, outdoor sports amenities, and playgrounds were closed. On May 10, 2020, it was announced that strict lowdown was being eased, with unlimited outdoor exercise being allowed from May 13, 2020. On July 4, further public amenities were reopened, including outdoor gyms and playgrounds. On July 25, 2020, indoor gyms and swimming pools reopened.

Figure 1 shows how the proportion of the sample in each physical activity category changed over the course of 22 weeks from the start of lockdown in March 2020. We observed a steady increase in the percentage of people who reported not having done any physical activity. The percentage of people who exercised for three hours or more also slightly increased in the first 10 weeks, but this then decreased and stablised. Notably, there is little evidence that the prevalence of physical activity as a whole showed sudden, concurrent changes with major adjustments in lockdown measures (as indicated by the vertical lines and dates in Fig. 1).

### Latent trajectory classes

**Figure 2:**
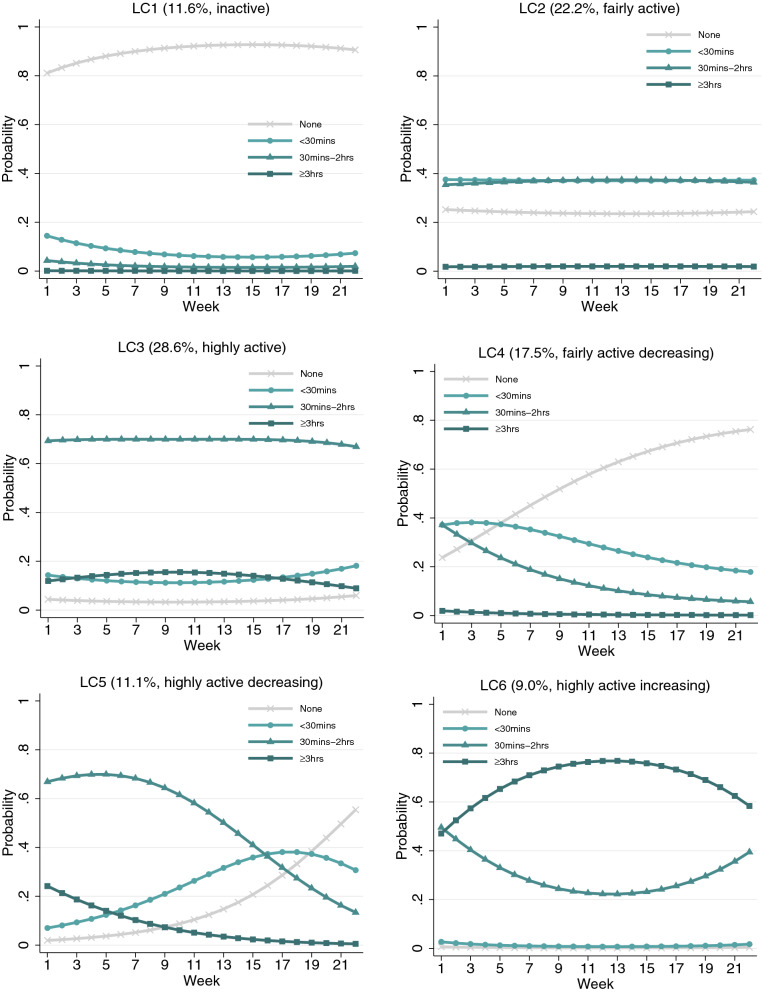
Estimated growth trajectories for different classes.

To determine the optimal number of latent trajectory classes, we estimated and compared across unconditional GMMs with different numbers of classes. Although the BIC and ABIC decreased with each additional class being added to the model, the ALMR-LR test of the seven-class GMM did not reject the six-class model (Table S1). Therefore, the six-class GMM model was chosen. It had an adequate quality of class membership classification (entropy = 0.72). The estimated probability of each physical activity category for each latent class (LC) is presented in Fig. 2.

The first three classes (LC1–3) were static, with little change observed over the 22-week period, making up 62.4% of the sample. The first class (LC1 = 11.6%) was marked by a high probability of physical inactivity (“inactive”). Participants in the second class (LC2 = 22.2%) had a moderate probability of doing physical activity for short (under 30 min) and medium (30 min to 2 h) durations (“fairly active”). The third class (LC3 = 28.6%) was the largest class, which consisted of people with a high probability of exercising for 30 min–2 h (“highly active”).

The last three classes (LC4–6) were dynamic, showing substantial changes over time. Both class four and five, together forming 28.6% of the sample, showed decreasing physical activity over time. The fourth class (LC4 = 17.5%) started from a moderate level of physical activity, very similar to LC2 when the lockdown started. In this class, the probability of being inactive increased rapidly over time, which was accompanied by declines in the short (< 30 min) and medium-duration (30 min–2 h) categories (“fairly active decreasing”). In contrast, participants in LC5 (11.1%) started from a high probability of exercising for 30 min–2 h, similar to LC3 at the beginning of lockdown. This probability was stable over the first few weeks but was followed by a rapid decline in subsequent weeks (“highly active decreasing”). This decline translated into an increased probability of being in the inactive or short duration (< 30 min) categories. The sixth class (LC6 = 9.0%) was the smallest and the most active, showing a high probability of exercising for medium (30 min–2 h) and long (≥ 3 h) durations. It was the only class that showed an overall increase in physical activity over time (“highly active increasing”). More specifically, the probability of exercising for a long duration (≥ 3 h) increased in the first 13 weeks, which was followed by a decrease when lockdown measures were substantially eased in June 2020. The growth trajectory of the long duration category (≥ 3 h) was the opposite of that for the category of medium duration (30 min–2 h), indicating exclusive transition between these two categories. Notably, this class had a very low probability of exercising for a short duration (< 30 min) or being physically inactive, which did not change over time.

### Factors associated with latent trajectory classes

We fitted a conditional GMM to examine how individual characteristics were related to class membership of physical activity trajectories, using LC1 (“inactive”) as the reference (Table 2). Young adults had higher odds of being “fairly active” (LC2) than people aged 30 to 45 (OR = 1.48, 95% CI 1.03–2.13), as did individuals with a degree or above compared to those with lower education (OR = 2.06, 95% CI 1.66–2.56). People living with children also had higher odds of being “fairly active” than those living alone (OR = 1.54, 95% CI 1.18–2.00). However, people with long-term physical (OR = 0.83, 95% CI 0.69–1.00) and mental (OR = 0.57, 95% CI 0.46–0.71) health conditions had lower odds of being “fairly active”.

**Table 2:** Results from the Growth mixture model with predictors of latent classes (LC) (LC1 as the reference, N = 35,915).

	LC2 (vs. LC1)		LC3 (vs. LC1)		LC4 (vs. LC1)		LC5 (vs. LC1)		LC6 (vs. LC1)	
	OR	95% CI	OR	95% CI	OR	95% CI	OR	95% CI	OR	95% CI
Woman (vs man)	1.02	[0.85–1.23]	0.96	[0.81–1.14]	1.19	[0.98–1.46]	1.19	[0.94–1.49]	**0.71**	**[0.58–0.87]**
Ethnic minority (vs white)	0.82	[0.57–1.17]	0.89	[0.62–1.29]	0.99	[0.66–1.48]	0.83	[0.52–1.31]	1.04	[0.68–1.59]
Age 18–29 (vs. 30–45)	**1.48**	**[1.03–2.13]**	1.24	[0.85–1.80]	1.32	[0.86–2.03]	**2.01**	**[1.33–3.04]**	**1.62**	**[1.02–2.57]**
Age 46–59 (vs. 30–45)	0.83	[0.67–1.03]	**1.32**	**[1.07–1.63]**	0.83	[0.65–1.06]	0.94	[0.72–1.22]	1.19	[0.89–1.59]
Age 60 + (vs. 30–45)	1.22	[0.93–1.59]	**2.23**	**[1.72–2.89]**	1.18	[0.87–1.60]	**1.75**	**[1.25–2.45]**	**2.07**	**[1.47–2.92]**
A-levels or equivalent (vs. GCSEs or below)	1.09	[0.87–1.36]	**1.25**	**[1.01–1.54]**	1.12	[0.87–1.43]	**1.44**	**[1.09–1.90]**	1.08	[0.83–1.41]
Degree or above (vs. GCSEs or below)	**2.06**	**[1.66–2.56]**	**2.36**	**[1.93–2.90]**	**1.35**	**[1.06–1.72]**	**1.78**	**[1.34–2.35]**	1.27	[0.97–1.68]
Household income < 30 k (vs ≥ 30 k)	0.82	[0.67–1.02]	**0.54**	**[0.44–0.65]**	0.80	[0.64–1.01]	**0.46**	**[0.36–0.60]**	**0.66**	**[0.52–0.84]**
Employed to unemployed (vs employed)	1.25	[0.90–1.73]	**1.68**	**[1.24–2.26]**	**1.52**	**[1.08–2.13]**	**1.79**	**[1.26–2.56]**	**1.77**	**[1.27–2.48]**
Unemployed/inactive (vs employed)	0.86	[0.69–1.08]	1.06	[0.87–1.29]	**0.77**	**[0.61–0.99]**	**0.70**	**[0.52–0.94]**	0.94	[0.72–1.21]
Living with others, but no children (vs alone)	1.20	[0.98–1.48]	**1.37**	**[1.13–1.67]**	1.04	[0.83–1.30]	**1.60**	**[1.21–2.11]**	**1.29**	**[1.01–1.64]**
Living with others, including children (vs alone)	**1.54**	**[1.18–2.00]**	**1.51**	**[1.17–1.95]**	1.28	[0.95–1.74]	**1.60**	**[1.13–2.26]**	**1.62**	**[1.17–2.23]**
Large town (vs city)	0.83	[0.65–1.04]	0.87	[0.69–1.09]	1.06	[0.81–1.38]	0.83	[0.61–1.13]	0.91	[0.67–1.22]
Small town (vs city)	0.92	[0.74–1.15]	1.03	[0.82–1.29]	0.93	[0.71–1.20]	0.91	[0.68–1.21]	1.00	[0.75–1.33]
Rural (vs city)	0.87	[0.67–1.12]	1.17	[0.92–1.48]	1.30	[0.97–1.74]	1.32	[0.97–1.80]	1.27	[0.96–1.67]
Long-term physical illness (vs none)	**0.83**	**[0.69–1.00]**	**0.49**	**[0.41–0.58]**	0.86	[0.70–1.04]	**0.55**	**[0.44–0.69]**	**0.61**	**[0.49–0.75]**
Long-term mental illness (vs none)	**0.57**	**[0.46–0.71]**	**0.48**	**[0.39–0.60]**	**0.70**	**[0.56–0.88]**	**0.49**	**[0.36–0.66]**	**0.55**	**[0.43–0.72]**

*LC1* inactive, *LC2* fairly active, *LC3* highly active, *LC4* fairly active decreasing, *LC5* highly active decreasing, *LC6* highly active increasing.

p < 0.05 in bold text.

Older adults had higher odds of being “highly active” (LC3) than those aged 30 to 45 (OR = 1.32–2.23), as did people with higher levels of education (OR = 1.25–2.36), those who lost their job (OR = 1.68, 95% CI 1.24–2.26), and people living with others (OR = 1.37–1.51). People from low-income households (OR = 0.54, 95% CI 0.44–0.65), and those with physical (OR = 0.49, 95% CI 0.41–0.58) and mental (OR = 0.48, 95% CI 0.39–0.60) health conditions had lower odds of being “highly active”.

For the dynamic classes, individuals with a degree or above (OR = 1.35, 95% CI 1.06–1.72) and those who lost their job (OR = 1.52, 95% CI 1.08–2.13) had higher odds of “fairly active decreasing” activity (LC4). People who were already unemployed or economically inactive at the start of lockdown (OR = 0.77, 95% CI 0.61–0.99) and those with mental health conditions (OR = 0.70, 95% CI 0.56–0.88) had lower odds of being in LC4.

Young adults under 30 (OR = 2.01, 95% CI 1.33–3.04) and older adults aged 60+ (OR = 1.75, 95% CI 1.25–2.45) had higher odds of “highly active decreasing” activity (LC5) than those aged 30–45. Also more likely to be in this class were people who: had higher levels of education (OR = 1.44–1.78); lost their job (OR = 1.79, 95% CI 1.26–2.56); and were living with others (OR = 1.60–1.60). Conversely, people who were from low-income households (OR = 0.46, 95% CI 0.36–0.60), unemployed or economically inactive (OR = 0.70, 95% CI 0.52–0.94), and had physical (OR = 0.55, 95% CI 0.44–0.69) and mental (OR = 0.49, 95% CI 0.36–0.66) health conditions had lower odds of being in LC5.

Finally, young adults under 30 (OR = 1.62. 95% CI 1.02–2.57), older adults aged 60+ (OR = 2.07, 95% CI 1.47–2.92), people who lost their job (OR = 1.77, 95% CI 1.27–2.48) and those living other others (OR = 1.29–1.62) had higher odds of “highly active increasing” activity (LC6). In contrast, women (OR = 0.71, 95% CI 0.58–0.87), people from low-income households (OR = 0.66, 95% CI 0.52–0.84) and those with physical (OR = 0.61, 95% CI 0.49–0.75) and mental (OR = 0.55, 95% CI 0.43–0.72) health conditions had lower odds of being in LC6, the only class where physical activity increased over time.

Given the similarities between LC4 (“fairly active decreasing”) and LC2 (“fairly active”), and between LC5 (“highly active decreasing”) and LC3 (“highly active”) at the beginning of the lockdown, we compared factors associated with these trajectories using alternative reference classes (Table S2). Individuals who had a degree or above (vs GCSEs or below), those who were unemployed or economically inactive (vs employed), and people aged 46–59 (vs 30–45) had lower odds of being in a class with decreasing physical activity. In contrast, people living in rural areas (vs cities) and aged 18–29 (vs 30–45) had higher odds of decreasing physical activity throughout the pandemic.

We carried out sensitivity analyses excluding keyworkers (n = 8651) who might have had a different experience during the lockdown due to still being able to go to work (analytical sample N = 27,264). The results were materially consistent with the main analysis, returning the same number of classes and very similar patterns of growth trajectories (Fig. S1). Other sensitivity analyses using piecewise growth models to reflect changes in lockdown measures also yielded similar results (Fig. S2).

## Discussion

This study is the first to examine the heterogeneity in longitudinal changes of physical activity during the COVID-19 pandemic. Building on recent longitudinal studies, which reported a general decline in physical activity at the start of the pandemic^14,26–31^, our analyses identified six unique classes of growth trajectories of physical activity. Three of these classes were stable, showing little change over time, including the inactive (11.6%), the fairly active (22.2%) and the highly active (28.6%). There were two classes showing declines in physical activity or increased physical inactivity, making up 28.6% of all participants. In contrast, 9% of participants showed an upward trend in physical activity over the observational period. These differing trajectories may explain the inconsistent findings to date from longitudinal studies testing whether physical activity returned to pre-lockdown levels with the easing of restrictions in the UK^26,27,30,31^.

This study further examined sociodemographic and health-related predictors of the patterns of physical activity growth trajectories. When comparing the three stable classes (inactive, fairly active, and highly active; LC1–3), we found no gender, ethnic, or urban/rural differences between them. However, people who were older, more educated, had a higher income, shared a household with others, and those without long-term physical and mental health problems, were more likely to be in a more active class. This is consistent with previous evidence that age, education, income, health status, and social support are associated with physical activity during lockdown^27,31,32,37–40,42,43^. In contrast, our findings are not consistent with prior evidence that women and people of non-white ethnicity were less active during lockdown^28,31,38–40,42,43^, but consistent with a review of reviews suggesting that gender and ethnicity are correlates but not determinants of physical activity^50^. Previous studies have also found that differences in physical activity between genders and ethnic groups are very small^4^.

A similar set of factors were found to predict the difference between the three dynamic classes (LC4–LC6) relative to the inactive (LC1). The dynamic classes were either fairly active or highly active at the start of lockdown, followed by decreases or increases in physical activity during lockdown. As with the stable classes, people who had higher education and income, lived with others, and did not have long-term health problems were more likely to be in the dynamic fairly or highly active classes than the inactive class. Additionally, individuals who became unemployed were more likely to be in the highly active or dynamic classes. It is possible that this group was unique in having to adjust how they spent their time during lockdown after becoming unemployed, compared to those who were employed or economically inactive throughout this period. Further research with this group may enable us to identify opportunities to increase physical activity and understand barriers that prevent this behaviour change^51^.

There were very few sociodemographic or health-related differences in the dynamic compared to stable classes that started at similar levels of physical activity (fairly active decreasing vs fairly active, and highly active decreasing vs highly active). However, those with decreasing levels of physical activity (i.e. in dynamic classes) were younger, more likely to be employed, living in rural areas, and had lower levels of education. Understanding why these factors were associated with decreasing physical activity is important for the development of interventions. Although younger people were generally more physically active before the pandemic^50^, several other studies have found that younger adults were more likely to report changes (both increases and decreases) in physical activity during lockdown than older adults^39,41,52^. This could be because younger adults were generally confined to smaller homes with no outdoor space and less space to exercise, meaning motivation to remain physically active reduced over time. Levels of physical activity in younger adults may also have decreased as restrictions eased and they replaced time previously spent on physical activity with more sedentary leisure activities, such as socialising. Additionally, being younger and having lower educational attainment are associated with higher levels of anxiety, depression, and loneliness during lockdown^53,54^, as well as increasing loneliness throughout lockdown^55^, all of which could have contributed to reductions in physical activity. It is also possible that decreasing physical activity was associated with being employed because individuals struggled to maintain levels of physical activity alongside working from home, decreased work-life balance, and increased stress and burnout throughout the lockdown^56,57^.

This study has a number of strengths including its large sample size, repeated weekly follow-up of the same participants over 22 weeks since the first UK lockdown, and robust statistical approaches. Although the UCL COVID-19 Social Study did not use a random sample, it does have a large sample size with wide heterogeneity, including good stratification across all major socio-demographic groups. In addition, analyses were weighted on the basis of population estimates of core demographics, with the weighted data showing good alignment with national population statistics and another large scale nationally representative social survey^53^. Despite all efforts to make our sample inclusive and representative of the adult population in England, we cannot rule out the possibility of potential biases due to omitting other demographic factors that could be associated with survey participation in the weighting process. Further, our analyses relied on self-reported time spent on physical activity which is subject to recall and reporting bias. We also lack data on people’s physical activity before the start of the COVID-19 pandemic, so it remains unknown how change or stability in physical activity observed in this study compare to usual levels in our sample before the pandemic. Finally, this study only has data during and post the first UK lockdown. Future studies could extend our analyses to explore whether, and to what extent, the longitudinal patterns of physical activity persist after the COVID-19 pandemic.

## Conclusions

Although there may have been a decline in physical activity in the general population at the start of the COVID-19 pandemic, it is important to acknowledge the heterogeneity in people’s longitudinal changes in physical activity. Our analyses have shown that over 62% of people experienced little change and another 9% increased their physical activity between March and August 2020. However, it should be highlighted that nearly 29% of people experienced reduced physical activity during the same period. Moreover, amongst the people with little change in physical activity, 12% were consistently inactive. Both of these groups call for attention and action. Given the well-established link between physical activity and health^1–5^, persistent or increased physical inactivity is likely to have both immediate and long-term implications for people’s physical and mental health, as well as general wellbeing ^58^. More public health efforts should be made to promote physical activity for the general population, and in particular for groups that are at a higher risk of inactivity or of reduced physical activity, during the COVID-19 pandemic and beyond.

## Supplementary Information

Supplementary Information.

## Data Availability

Anonymous data will be made publicly available following the end of the pandemic.

## Abbreviations

ABIC: Sample-size adjusted Bayesian information criterion
ALMR-LR: Adjusted Lo-Mendell-Rubin likelihood ratio
BIC: Bayesian information criterion
CI: Confidence interval
COVID-19: Coronavirus disease 2019
GCSE: General certificate of secondary education
GMM: Growth mixture modelling
LC: Latent classes
LMR-LR: Vuong-Lo-Mendell-Rubin likelihood ratio
OR: Odds ratio
UCL: University College London
UK: United Kingdom
