# Supplementary material for: Longitudinal changes in physical activity during and after the first national lockdown due to the COVID-19 pandemic in England
Source: Sci Rep. 2021 Sep 2;11:17723. doi: 10.1038/s41598-021-97065-1 (PMC8413348; doi:10.1038/s41598-021-97065-1)
Supplement: Supplementary file 1 — Supplementary Information. [file 41598_2021_97065_MOESM1_ESM.docx]

Supplement Material

Table S1 Model fit indices for different model specifications

| Model specification | BIC | ABIC | LMR-LR | ALMR-LR | Entropy |
| --- | --- | --- | --- | --- | --- |
| 1-class GMM | 1,098,424 | 1,098,408 | NA | NA | NA |
| 2-class GMM | 947,350 | 947,322 | <0.001 | <0.001 | 0.855 |
| 3-class GMM | 903,370 | 903,329 | <0.001 | <0.001 | 0.857 |
| 4-class GMM | 874,221 | 874,167 | <0.001 | <0.001 | 0.814 |
| 5-class GMM | 867,525 | 867,459 | <0.001 | <0.001 | 0.765 |
| **6-class GMM** | **862,970** | **862,890** | **<0.001** | **<0.001** | **0.723** |
| 7-class GMM | 860,626 | 860,534 | 0.199 | 0.209 | 0.728 |

Notes: Models with smaller BIC and ABIC have a better fit. LMR-LR and ALMR-LR compare model fit between models with k classes and (k − 1) classes. A significant p value indicates a significant model fit improvement in the k-class model. Entropy is a measure of the quality of class membership classification. A value of 0.80 is considered as high, 0.60 is medium, and 0.40 is low

Table S2. Results from the Growth mixture model with predictors of latent classes (LC) using alternative reference class (N=35,915)

|  | LC4 (vs. LC2) | | LC5 (vs. LC3) | | LC5 (vs. LC4) | | LC6 (vs. LC3) | |
| --- | --- | --- | --- | --- | --- | --- | --- | --- |
|  | OR | 95% CI | OR | 95% CI | OR | 95% CI | OR | 95% CI |
| Woman (vs man) | 1.17 | [0.96-1.41] | 1.24 | [1.01-1.52] | 0.99 | [0.80-1.24] | **0.74** | **[0.62-0.88]** |
| Ethnic minority (vs white) | 1.21 | [0.81-1.80] | 0.92 | [0.58-1.47] | 0.84 | [0.54-1.30] | 1.16 | [0.78-1.73] |
| Age 18-29 (vs. 30-45) | 0.89 | [0.61-1.29] | **1.63** | **[1.10-2.39]** | **1.52** | **[1.06-2.19]** | 1.31 | [0.87-1.97] |
| Age 46-59 (vs. 30-45) | 1.00 | [0.79-1.25] | **0.71** | **[0.56-0.91]** | 1.14 | [0.89-1.46] | 0.90 | [0.69-1.17] |
| Age 60+ (vs. 30-45) | 0.97 | [0.74-1.28] | 0.79 | [0.58-1.07] | **1.48** | **[1.07-2.04]** | 0.93 | [0.69-1.26] |
| A-levels or equivalent (vs. GCSEs or below) | 1.03 | [0.80-1.32] | 1.15 | [0.88-1.51] | 1.29 | [0.99-1.68] | 0.86 | [0.68-1.10] |
| Degree or above (vs. GCSEs or below) | **0.65** | **[0.52-0.82]** | **0.75** | **[0.57-0.98]** | 1.32 | [1.00-1.74] | **0.54** | **[0.42-0.69]** |
| Household income <30k (vs ≥30k) | 0.97 | [0.79-1.20] | 0.86 | [0.68-1.08] | **0.57** | **[0.45-0.73]** | 1.23 | [1.00-1.52] |
| Employed to unemployed (vs. employed) | 1.21 | [0.89-1.66] | 1.07 | [0.79-1.44] | 1.18 | [0.87-1.60] | 1.06 | [0.81-1.38] |
| Unemployed/inactive (vs. employed) | 0.89 | [0.70-1.15] | **0.66** | **[0.50-0.87]** | 0.90 | [0.67-1.22] | 0.88 | [0.70-1.11] |
| Living with others, but no children (vs alone) | 0.86 | [0.69-1.07] | 1.17 | [0.89-1.53] | **1.54** | **[1.17-2.03]** | 0.94 | [0.75-1.16] |
| Living with others, including children (vs. alone) | 0.84 | [0.64-1.10] | 1.06 | [0.76-1.47] | 1.25 | [0.90-1.73] | 1.07 | [0.81-1.42] |
| Large town (vs. city) | 1.28 | [0.99-1.65] | 0.96 | [0.71-1.28] | 0.79 | [0.58-1.06] | 1.04 | [0.80-1.37] |
| Small town (vs. city) | 1.01 | [0.80-1.26] | 0.88 | [0.67-1.16] | 0.98 | [0.74-1.29] | 0.97 | [0.76-1.25] |
| Rural (vs. city) | **1.50** | **[1.15-1.95]** | 1.13 | [0.86-1.49] | 1.02 | [0.76-1.37] | 1.08 | [0.86-1.36] |
| Long-term physical illness (vs. none) | 1.03 | [0.85-1.25] | 1.12 | [0.90-1.39] | **0.64** | **[0.52-0.80]** | **1.24** | **[1.02-1.50]** |
| Long-term mental illness (vs. none) | 1.23 | [0.97-1.56] | 1.01 | [0.72-1.42] | **0.69** | **[0.51-0.94]** | 1.15 | [0.88-1.50] |

Note: p<0.05 in bold text. LC1: inactive, LC2: fairly active, LC3: highly active, LC4: fairly active decreasing, LC5: highly active decreasing, LC6: highly active increasing.


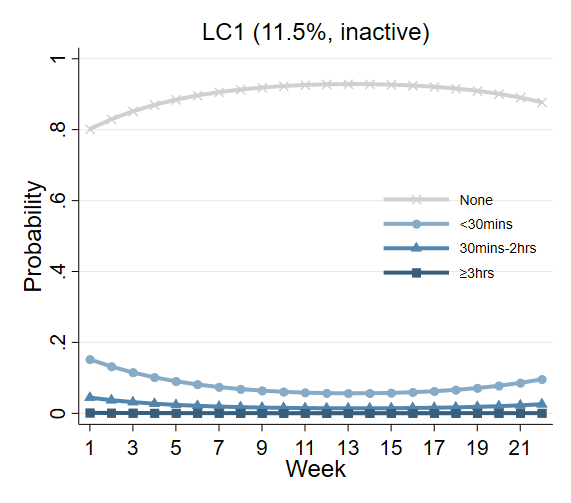

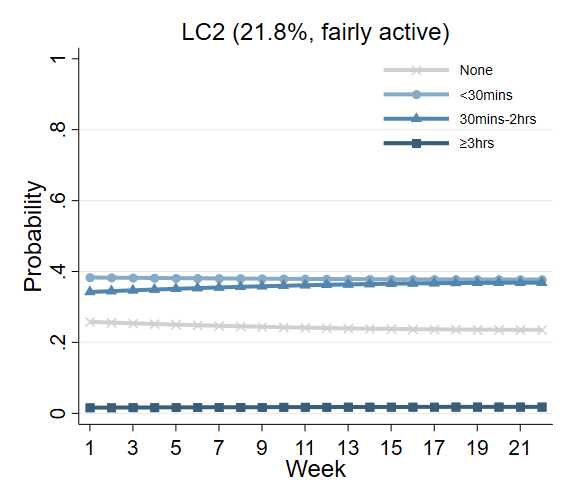

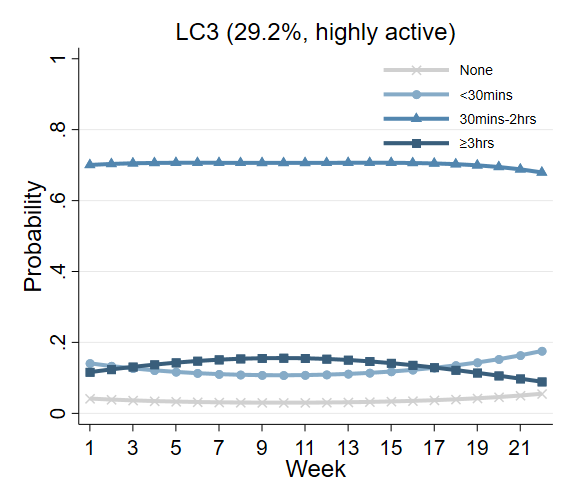

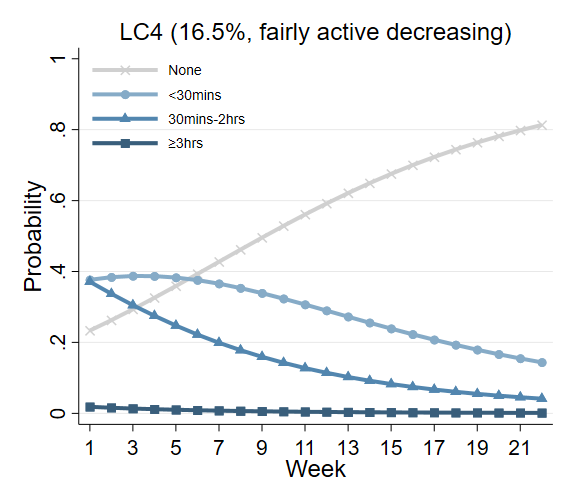

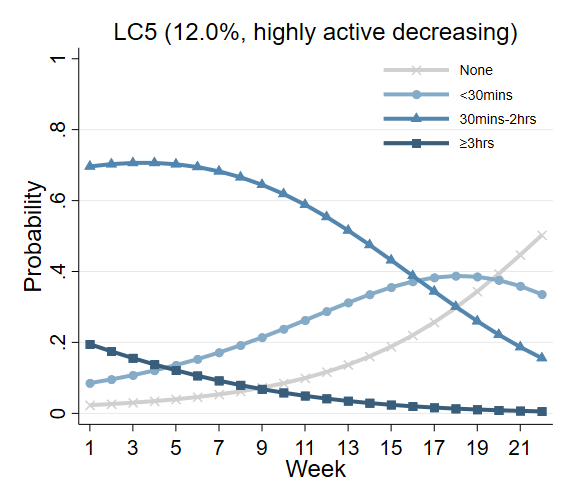

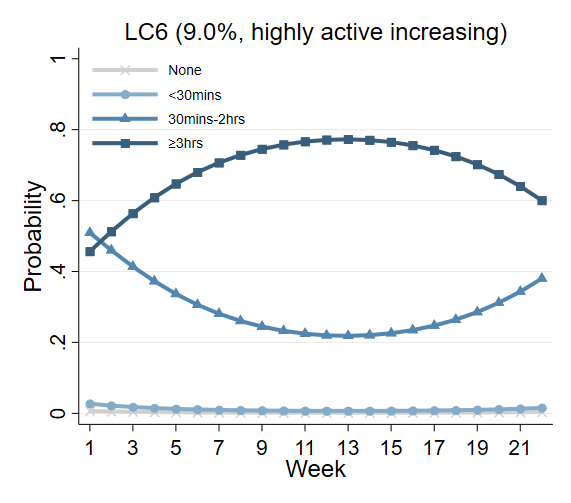


Figure S1. Estimated growth trajectories for different classes (excluding keyworkers)


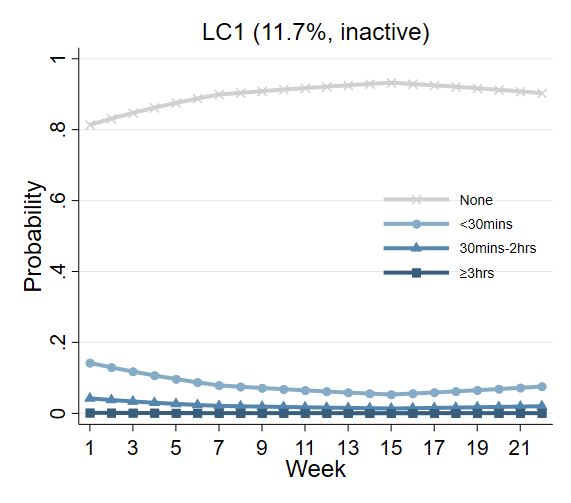

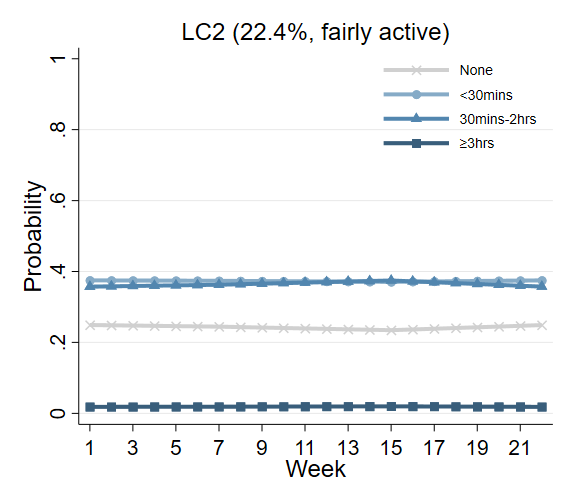

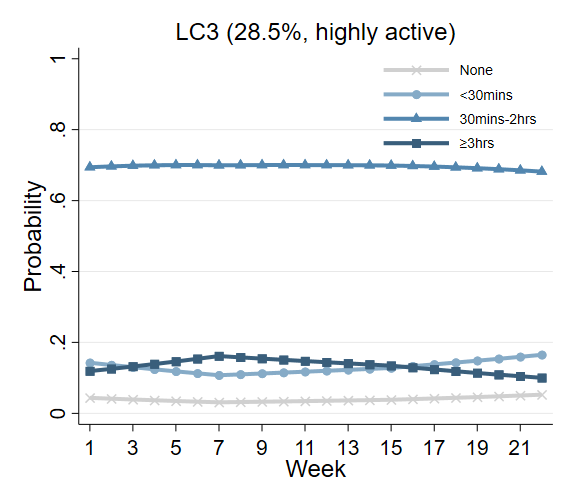

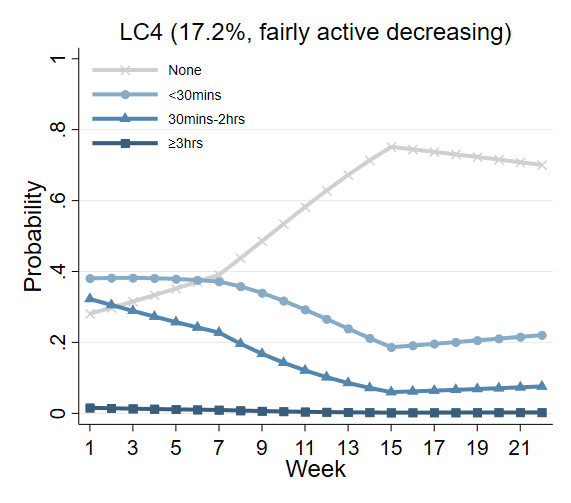

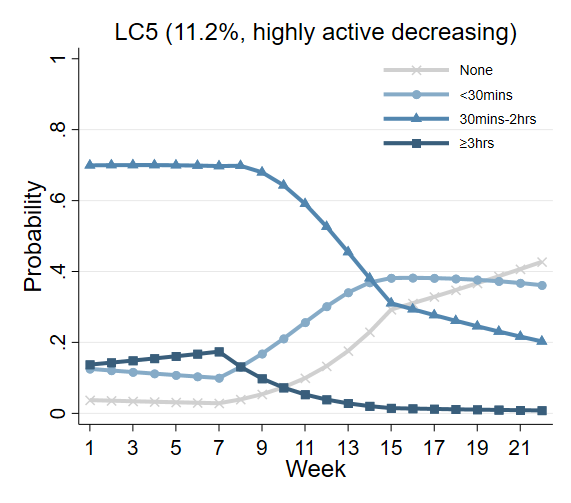

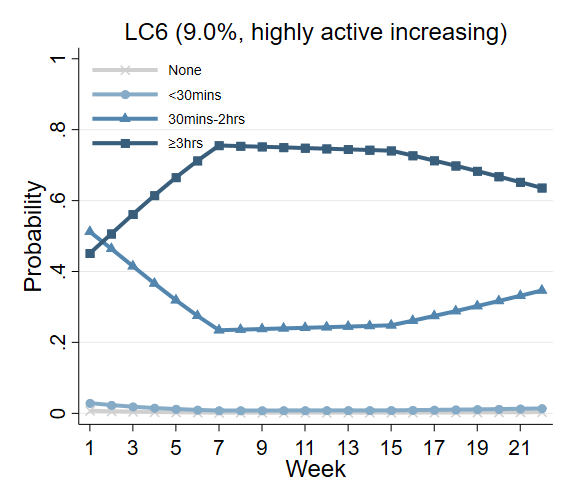


Figure S2. Estimated growth trajectories for different classes (piecewise growth)
